# Running a research group in the next generation: combining sustainable and reproducible research with values-driven leadership

**DOI:** 10.1093/jxb/erac407

**Published:** 2022-12-19

**Authors:** Jacqueline Monaghan, Siobhan M Brady, Elizabeth S Haswell, Sonali Roy, Benjamin Schwessinger, Heather E McFarlane

**Affiliations:** Department of Biology, Queen’s University, Kingston ON, Canada; Department of Plant Biology and Genome Center, University of California at Davis, Davis CA, USA; Department of Biology, Washington University in St Louis, St Louis MO, USA; College of Agriculture, Tennessee State University, Nashville TN, USA; Research School of Biology, Australian National University, Canberra, Australian Capital Territory, Australia; Department of Cell and Systems Biology, University of Toronto, Toronto ON, Canada

**Keywords:** Grant writing, inclusion, lab management, leadership, mentorship, reproducibility, research vision, values statement

## Abstract

In the summer of 2021, we held a community workshop at the International Congress of Arabidopsis Research (ICAR) aimed at early career researchers and focused on values-based lab leadership. Here, we elaborate on ideas emerging from the workshop that we hope will allow current and future group leaders to reflect on and adjust to the rapidly evolving nature of the academic scientific enterprise.

## Emerging challenges faced by new academic group leaders

New group leaders, including principal investigators, assistant professors, and anyone else who recently began leading an independent research team, are faced with an array of challenges. Many of these challenges are associated with an expansion of responsibilities and expectations as scientists become group leaders. Group leaders must identify, define, answer, and communicate research questions and results; secure, administer, and track funding; and recruit, train, supervise, and mentor lab personnel. These tasks are usually overlaid with other responsibilities including teaching, committee service, public outreach, and more. Thankfully, most new group leaders have access to mentorship and support from colleagues, staff, and others who can help them navigate this substantial career transition. However, some new group leaders are facing these challenges alone. On top of it all, the face of science is constantly changing. Funding mechanisms, interdisciplinary research, the nature of student training, attitudes towards leadership, and modes of research communication have all undergone significant shifts in the last decades. While new group leaders usually have excellent mentors for traditional topics, everyone is facing these new developments in the research landscape.

Here, we aim to provide some guidance for new group leaders on a subset of these challenges, based on a virtual workshop held at the International Congress of Arabidopsis Research (ICAR) during the summer of 2021 entitled: Running a Research Group in the Next Generation. Inspired by questions such as: ‘What does it mean to lead a research program?’, ‘How can we make sustainable investments of our time, energy, and focus?’, and ‘What does it mean to advance knowledge?’, we have collected insights, best practices, and resources from the workshop at ICAR 2020/2021.

## Developing a vision for a sustainable research program

Post-doctoral research is focused on completion of one or several projects that culminate in peer-reviewed publications. Time as a post-doctoral scholar is spent gaining diverse technical skills and honing expertise in a field of research. However, a new set of skills is needed for group leaders to succeed. How does one transition from a researcher who is busy doing and interpreting to one who generates a research vision and fully fledged research program for their independent group? Two complementary approaches that have worked well for us are: (i) to identify a problem or question that is intrinsically exciting and develop a research program around it; and/or (ii) to identify sources of funding, and then consider how to develop a research program that addresses the goals of that specific funding agency. Of course, these are not mutually exclusive, and any solid research program combines elements of personal passion and more practical elements such as funding (Lai, 2022).

**Fig. 1.**
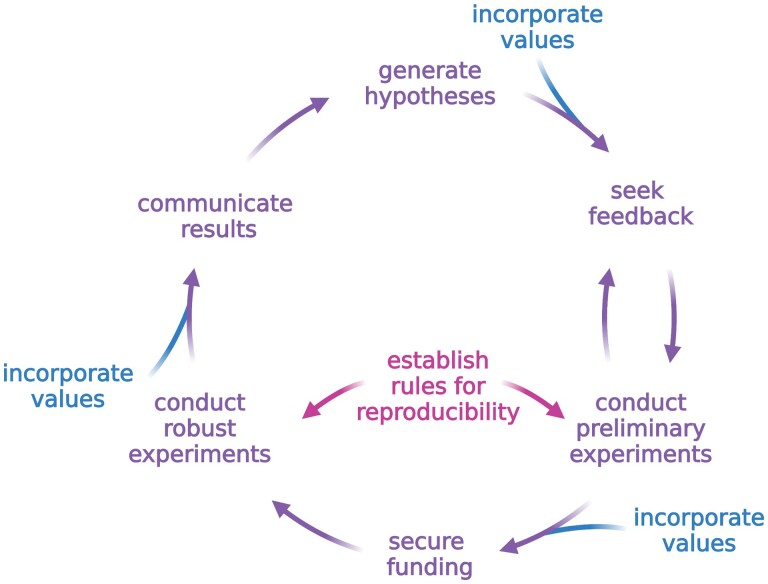
Developing a vision for a sustainable research program. Developing a research vision is an iterative, cyclical process. Throughout the process, group leaders can establish systems for reproducibility to ensure that sound conclusions are being drawn. At each stage that requires prioritization, group leaders can prioritize using their values. For example, when deciding which possible research project to pursue, a new group leader who strongly values community or teamwork might prioritize seeking funding for a project that involves a citizen science component, while a new group leader who values innovation might prioritize securing funding for a project with a strong potential for technology development. This figure was created with BioRender.com.

Future group leaders can begin crafting a research vision even before they open their new labs (Spencer *et al.*, 2022). While progressing through post-doctoral research projects, future group leaders can periodically assess the larger biological questions for which they have passion. It can be difficult to find the time to pause in the midst of all the experiments and analyses, and to consider maintaining an avid interest in a topic over the next 5–10 years, but this can be a rewarding experience. Taking the time to understand why one is undertaking a research project can help to maintain enthusiasm and curiosity over time, and can help to guide the how and what of future research projects (Sinek, 2011). The process of establishing a research vision is iterative and should be guided by critical and honest feedback from peers and senior colleagues. Constructive feedback can help new group leaders refine their vision by incorporating others’ opinions on logic and feasibility. This cycle of review and reformulation of a research vision is fundamental to crafting a consistent research program with carefully considered risks and benefits (Fig. 1).

Securing external funding is essential to sustain a fruitful research program and training environment. Group leaders should investigate local, regional, national, and international research funding opportunities available to them via federal or other funding agencies. What types of projects do organizations fund? How long are funding cycles and when are the key deadlines? What attributes of a project make it competitive for funding? When writing a grant, formulate ideas into independent aims, ideally two to three, with hypotheses clearly delineated, the methods outlined, and expected outcomes listed. Writing good grants is an iterative process that ideally incorporates feedback from multiple readers prior to submission. Institutes and universities typically have dedicated research support offices that can provide assistance with grant writing, compliance, and budgeting— investigate which grant writing services are available, and take advantage of them. Similarly, some granting agencies will offer direct advice on applications. Determine if there are any preliminary data that could demonstrate that this new research program has the means to succeed, and which can serve as a nucleus for the first publications as a new group leader. In addition, seed grants are available which do not require extensive preliminary data and are meant to initiate research programs; equipment grants are available to support essential infrastructure; and some funding opportunities are available exclusively for pre-tenure faculty or to help build new collaborations.

Many new group leaders have several exciting ideas for research projects, but it is often advisable to start with a focused research vision when funding is limited and new personnel are being trained. It is valuable to consider how these different research projects align with the lab’s mission, because this can help new group leaders focus their research vision to make substantial progress on their first funded project. Similarly, it is important to exercise caution when fitting a proposal to a specific funding scheme that deviates from the established research vision (Lai, 2022). Funding agencies typically assess: (i) the fit of the research question to that outlined in the funding announcement; (ii) the importance of the research question; (iii) the quality of the research project including innovation, its transformative nature, feasibility, time scale, risk analysis, and contingency plans; and (iv) the track record of the applicant and/or collaborators. Funded proposals where the research question does not match the core ideas outlined in your lab’s research vision can lead to dissatisfaction which can spill over into lab culture.

## Values-driven leadership and establishing lab culture

A values-driven approach to leading a lab can empower both new and experienced group leaders to define how to focus their time and effort on what matters to them; feel good about making difficult choices about where to deploy their time, energy, and funding; and help foster an inclusive and equitable lab environment. Values affect lab culture, training environment, and research approach, and they also influence how success is defined for a research team. Articulating values allows deliberate influence on one’s leadership style and lab culture—this awareness of what can be influenced and why is powerful knowledge.

**Fig. 2.**
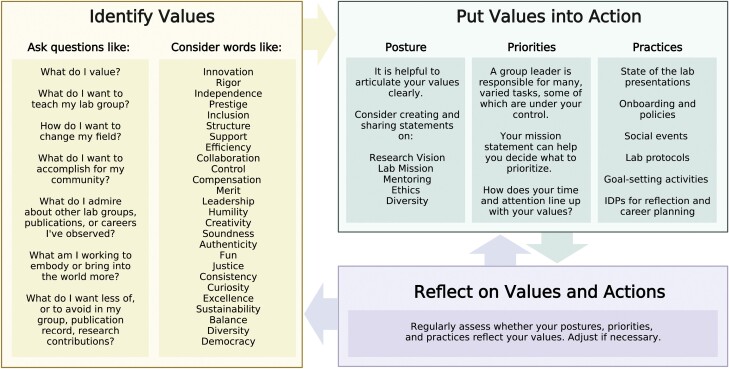
Establishing a value-based approach to leadership in research. Iterative cycling through identifying values, putting them into action, and determining whether your actions reflect your values can transform a new group leader’s sense of purpose and agency. The steps are: (i) identify your values; (ii) formalize them into postures; (iii) use them to set your priorities; (iv) put them into practice; and, finally, (v) evaluate the outcomes and adjust as needed. Readers are encouraged to search for expanded lists of values to reflect upon (several are available online). This figure was created with BioRender.com.

Values are a person’s principles or standards of behavior; one’s judgment of what is important in life (Tiberius, 2018). They are different from goals, which are aims or desired results. Goals are distinct from values, and should be derived from them, not the other way around. It is important to recognize that everyone in a lab has their own values and their own goals, and only some of these will overlap with those of the group leader. We encourage new group leaders to identify their own values and to consider holding an annual or biannual lab meeting where all members of the lab do so as a group activity. Identifying values is not a typical activity for scientists in training, so we have put together a worksheet on one possible process (Fig. 2).

Importantly, values are just concepts until they are articulated through actions. In our view, values can be used to establish three things: posture, priorities, and practices. Posture means a particular way of dealing with or considering something; an approach or inner attitude. One way to formalize a values-driven posture is to craft a statement. For example, mission statements or diversity statements can help articulate established values and goals.

Once clearly stated, our values can guide how we prioritize time, energy, and finances. All group leaders face a dizzying list of tasks, and it is not possible to do all of them perfectly (Haswell, 2017). Aligning time and attention with values can help identify the things most important to the research group, and ensure that time and energy are allocated accordingly. By keeping a regularly updated list of top priorities in a prominent place, new group leaders can track and focus on key goals that are aligned with their personal values (Fig. 2). For example, if ‘supporting lab members’ is a top priority on your list and a student asks you to review an abstract for a meeting, you would probably make time for that as soon as possible. Whereas, if ‘administrative service’ is a lesser priority on your list and you are asked to serve on another committee, you might decide to only do so if you are already meeting your obligations in other higher ranked values. Furthermore, understanding individual work rhythms and organizing projects with those rhythms in mind is key to effective time management that can help multi-tasking group leaders. Consider frameworks such as time blocking (Hakoune 2022), the Quadrant Method (Nguyen, 2012), or the Shuffle (Kamoun, 2022) when learning to set reasonable goals and prioritize work.

There are regular practices that can help new group leaders stay aligned with their values. State of the lab presentations, social events, lab protocols and policy documents or wikis, lab goal-setting activities, and individual development plans (IDPs) (Hobin *et al.*, 2012; Klasek, 2019) for reflection and career planning all serve to formalize and externalize values. These can be implemented into a yearly lab calendar, which can help group leaders incorporate these practices into their lab culture in a steady and predictable way. Because values-driven leadership is a cyclic and iterative process and values may change over time, ongoing reflection is necessary. For example, consider whether values are reflected in the outcomes from the lab. If not, adjust posture, priorities, or practices (Fig. 2). Honesty is important—we are all human, we all make mistakes, but we must be humble enough to learn from mistakes and grow.

Group leaders can encourage lab members to share their individual values and work together to formulate outward-facing postures that reflect the group’s values, such as a lab code of conduct or a lab diversity statement. These exercises can improve communication, promote shared understanding, encourage teamwork amongst the group, and establish a lab culture of collaboration and cooperation. Furthermore, commonly established and agreed-upon codes of conduct can set standards for acceptable behavior in the group such as mutual respect, teamwork, and transparent communication.

## Establishing standardized workflows for reproducibility

**Fig. 3.**
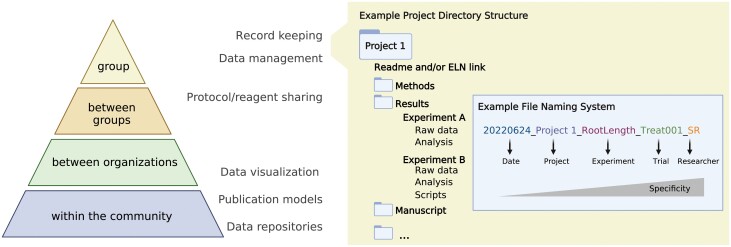
Establishing best practices for reproducibility. New group leaders are ideally positioned to build systems for reproducibility into their research programs. Key opportunities to improve reproducibility exist when sharing research at different levels, such as within the group, between groups or organizations, and with the community. For example, systematic file naming can enhance research sharing within a research group. This figure was created with BioRender.com.

Reproducibility and transparency in research should be guiding principles in science. However, almost 70% of surveyed researchers report having trouble replicating either their own findings or those of their peers (Baker, 2016). New group leaders are ideally positioned to adopt tools and establish systems to increase reproducibility in their labs, which can reduce time and resources spent on replicating data. In addition, improving reproducibility helps the wider scientific community by ensuring other labs are able to replicate findings and build research upon those findings. Key tools for reproducibility include those aimed at better record keeping, project management, data sharing, and dissemination within a research group, between groups, between organizations, and within the larger scientific community (Fig. 3). There are a lot of digital project and lab management tools available to help organize multiple projects that are quite effective (Powell, 2021). Scientific inquiry is increasingly a collaborative endeavor, and ensuring that other members of the community are able to replicate experiments and findings is essential for rapid research progress, especially within large, multi-lab projects. Without organized resources within a lab, sharing data, protocols, and material in a timely manner can be difficult.

Students and early career scientists come from different backgrounds and may have little to no training in best practices for data collection. Some of the easiest practices group leaders can initiate to improve reproducibility within their group include standardizing rules for file naming and organization, and training students on how to maintain a lab notebook. Lab files should be available on a shared drive, accessible by at least the trainee and the group leader, and should be backed up regularly. A good file name is both human and machine readable, and has sufficient detail for easy retrieval by a simple search (Fig. 3). These features allow group leaders to curate and access data generated by anyone in their team, past or present. Similarly, folder structure can be organized by the name of the funding organization (NSF-REPRO), data type (manuscripts, phenotyping raw data, codes), and file type, version (e.g. NSF-REPRO>Manuscripts>JXB-paper>V1). For all experimental data, raw and metadata files must be included. Authors are now expected by various funding organizations and journals, including here at the *Journal of Experimental Botany*, to make their raw data freely available (National Institutes of Health, 2020). Each lab can develop a set of file/folder naming rules that are appropriate for the type of research being conducted; the specifics of the file naming rules should be determined by the group leader and followed by all lab members. It is important to clearly communicate these expectations to trainees during the ‘onboarding’ phase, which is the initial period during which new hires are trained in shared lab practices.

Undergraduate instruction in maintaining laboratory notebooks varies between institutions and labs, and extensive note taking is not natural to many individuals. Historically, scientific records have been maintained manually in paper laboratory notebooks, which does not always meet current needs to text-search or include attachments with raw data in electronic formats. Electronic laboratory notebooks (ELNs) satisfy a number of these requirements, but, just as with paper lab notebooks, new trainees must be instructed on note taking standards and provided with examples for clarity. Group leaders are encouraged to explore free and paid ELN options (Harvard Longwood Medical Area Research Data Management Working Group, 2021) and choose one that suits their budget and needs, including requirements for intellectual property protection such as timestamping and archive functions. Group leaders who ensure that trainees have adopted these rules via regular check-ins are more likely to ensure that these best practices for reproducibility are established and maintained.

A second set of reproducibility checkpoints pertain to data sharing with colleagues and collaborators (Fig. 3). There are two practices that are recommended—sharing protocols and sharing reagents. Several platforms exist for standardized protocol writing (for example, Protocols.io, Bio-protocol, PLOS ONE Lab, and Study Protocols). As a starting point, lab members should be instructed to add as much information as feasible for each step in a protocol. For example, including the purpose of an experiment provides context, and including photos or diagrams and time intervals for each stage promotes experimental replicability. Include manufacturer, catalog number, and batch number for reagents, as there may be minor to significant variations among manufacturers and lots. In general, a good protocol is one that can be considered an independent publication (Baker, 2021; Daudi, 2022). Sharing reagents can be accomplished through general molecular biology repositories, such as addgene or plasmids.eu, and organism-specific repositories, such as TAIR, uNASC, MaizeGDB, and other regional stock centers, where the responsibility for maintaining and distributing these resources rests with dedicated personnel who charge a minimal distribution fee. When appropriate, raw data and analysis code should be deposited in data and code repositories such as Github/Zenodo, NCBI, PRIDE, or similar, and this is increasingly becoming a requirement for publication in peer-reviewed journals.

The third tier of establishing reproducible lab practices (Fig. 3) involves defining standards for transparent science communication, whether to academic colleagues, policymakers, or members of the general public. Lab members should be educated on effective methods of data visualization and given guidance on where and how their findings can be shared to reach a diverse audience. Before data are published, it is important to consider the most transparent method of data visualization for accessibility to a wide range of audiences (Weissgerber *et al.*, 2015). Many new trainees, for instance, may be used to seeing data presented in the form of bar graphs, even though such graphs are not always the most informative when it comes to presenting continuous data. It is important to teach trainees the appropriate statistical analyses to use and to assess sample size, data distribution, and whether data are continuous or discrete.

Reproducible science is integral to open science, which advocates for research to be made available to the scientific community and the general public without paywall barriers. This enables the free use of publicly funded research information by anyone. While an increasing number of journals are adopting open access publishing options, advancements in publishing are not restricted to this area. New group leaders should keep an eye out for ever-changing and new publication models for their datasets. For example, the ‘registered report’ article type (which has recently been adopted by several journals, including *Plant Direct*) allows researchers to publish a peer-reviewed research plan, followed by a manuscript describing the results. Publish, then review models (as adopted by *eLife*; Eisen *et al.*, 2020) involve making manuscripts openly available as preprints before peer review.

## Perspectives for the future

The unique challenges faced by early career researchers and new group leaders have gained substantial visibility in recent years (Pain, 2018). Excellent new programs are providing much needed support for this important career transition (for example, the National Center for Faculty Development and Diversity, New PI Slack, Making the Right Moves, EMBO Lab Management Courses, Plant Postdocs, and Plantae). Developing a coherent research vision can help new group leaders attract and prioritize funding to support a sustainable research program. Values-driven leadership can help new group leaders consciously and wisely invest their time and energy as they balance their new responsibilities. Developing frameworks for reproducible and open science will make research results more widely accessible and support the rapid advancement of science.

As new group leaders spend time assessing their leadership, mentoring, and lab management paradigms, they can also contemplate how they would like to be mentored. This should be an active process. In addition to formal mentoring programs that may be in place within a department or institution, consider seeking out other mentors who may foster a supportive relationship, provide an objective viewpoint on research and funding opportunities, paths towards tenure or job security, as well as the multitude of questions that can arise when determining how best to mentor trainees. As no one person is an expert in all of these areas, it is important to establish a framework for professional support and mentorship to foster a successful career that includes physical and mental well-being.

Beginning and continuously leading a research group can be overwhelming, often coming with an untenable list of tasks to perform and both an internal and external pressure to succeed. Mental health initiatives are normalizing conversations about the challenges we face in and outside of the lab. Prioritizing health by accessing this support will help new group leaders implement work–life boundaries and can enable a sustainable research career. In addition, diversity, equity, and inclusion efforts and anti-racism initiatives are tackling systematic barriers to participation and thriving in academia. New group leaders are particularly encouraged to engage in these initiatives to help shape workplaces and support their future colleagues.
